# Development of a preoperative questionnaire to improve satisfaction with hallux valgus repair: A Delphi study

**DOI:** 10.1371/journal.pone.0276303

**Published:** 2022-10-24

**Authors:** Cédric Blouin, François Genet, Philippe Denormandie, Wilfrid Graff, Antoine Perrier

**Affiliations:** 1 UFR Simone Veil-Santé, UR2020 Erphan, Université Versailles Saint-Quentin-en-Yvelines (UVSQ), Montigny-le-Bretonneux, France; 2 Service de Chirurgie Orthopédique, Hôpital de la Croix-Saint-Simon, Groupe Hospitalier Diaconesses–Croix-Saint-Simon, Paris, France; 3 ISPC Synergies, Paris, France; 4 Département PARASPORT-SANTE, Unité Péri Opératoire du Handicap, (UPOH-Perioperative Disability Unit), Service de Médecine Physique et de Réadaptation, Hôpital Raymond-Poincaré, Groupe Hospitalo-Universitaire APHP-Université PARIS SACLAY, Garches, France; 5 UFR Simone Veil-Santé, END: ICAP, Inserm U1179, Université Versailles Saint-Quentin-en-Yvelines (UVSQ), Montigny-le-Bretonneux, France; 6 Service de Chirurgie Orthopédique, Hôpital Raymond Poincaré, APHP, Garches, France; 7 Groupe Mutuelle Nationale des Hospitaliers (MNH), Paris, France; 8 Laboratoire de Recherche Translationnelle et D’Innovation en Médecine et Complexité TIMC, CNRS, Grenoble, France; 9 Service de Diabétologie, Groupe Hospitalier Pitié-Salpêtrière, Paris, France; Baqai Medical University, PAKISTAN

## Abstract

**Background:**

Satisfaction with hallux valgus repair is often poor, despite good surgical outcomes. Many tools have been developed to assess the outcome of the procedure; however none evaluate the association between the initial motive for repair and the reasons for post-surgical dissatisfaction. The aim of this study was to develop a new tool to analyse the subjective and objective expectations of individuals during a pre-operative consultation for hallux valgus repair in order to improve post-surgical satisfaction.

**Methods:**

We first collected the reasons for dissatisfaction with repair from the medical files of dissatisfied individuals. Then, a steering committee of 4 French experts in the management of hallux valgus designed a questionnaire based on the reasons for dissatisfaction. We then used the DELPHI method to validate the questionnaire: we submitted the questionnaire to a panel of 34 francophone experts in hallux valgus repair for rating in 4 rounds.

**Results:**

The medical files of 853 individuals were reviewed and a 52-item questionnaire relating to expectations from hallux valgus surgery was drafted. After the 4 rounds, a final 44 item questionnaire reached consensus. Thirteen items related to clinical and psychological profile, 5 to pain, 9 to physical activity, 4 to aesthetics and 13 to footwear.

**Conclusion:**

This tool should facilitate gathering of individuals’ expectations from hallux valgus repair to ensure realistic goals and reduce post-surgical dissatisfaction.

## Introduction

Hallux valgus is the most common disorder of the foot [1]. It affects 30% of women and 13% of men of all ages, and its prevalence increases with age [2]. The only effective treatment for hallux valgus is surgery [3–5].

Hallux valgus deformity is commonly analysed descriptively. The clinical examination of this deformity begins with an exhaustive morpho-static and morpho-dynamic, bilateral analysis of the lower limbs, associated with muscular, articular, vascular, neurological, etc. assessments [6–8]. The extent of the hallux valgus can be graded clinically with the Manchester scale, which is a method of assessing the deformity using a set of standardised photographs [9].

In order to make a therapeutic decision, the clinical assessment must be complemented by imaging. Metrology using standard radiographs of the foot and ankle, performed under load, must be comparative. A large number of angular measurements of the foot is necessary to understand the pathophysiology of the deformity [10–12]. Measurements of the hallux valgus angle (HVA) and the intermetatarsal angle (IMA) allow classification of the severity of the hallux valgus [13, 14]. In addition, the anatomical characteristics of the sesamoid bones of the hallux can be evaluated with dorsoplantar and axial radiographs. They allow morphological classification and measurements such as the sesamoid rotation angle (SRA), which is important for the choice of surgical technique [15–20].

Recently, a new classification of hallux valgus, based on three-dimensional anatomy of the first ray, with a decision algorithm for management has been proposed [21]. Radiological measurements are nowadays often complemented by three-dimensional CT techniques [22–24].

Baropodometry is also used as part of the routine clinical assessment of hallux valgus [25–27]. In particular, it provides a measurement of dynamic plantar pressures before and after the operation [28, 29]. However, none of these assessments provide information about the individual’s functional goal for surgery.

Postoperative satisfaction depends on the adequacy between the result obtained and the individual’s preoperative expectations [30, 31]. However, studies have shown that there is no correlation between subjective satisfaction and deformity reduction after hallux valgus repair [32–34]: the criteria for a good outcome differ between the surgeon and the individual [35]. Despite adequate deformity reduction following surgery [32, 36], dissatisfaction with the outcome is frequent [31, 37]. We suggest this is because the focus of the preoperative consultation is typically on pain, and the individual’s needs and expectations are not fully expressed and/or are not fully investigated by the surgeon. It has been reported that clinicians only question people regarding their motive for repair and their postsurgical expectations in 36% of cases [38]. When they do ask, they interrupt the response in 70% of cases after a mean 11 s [38]. This short time could explain why the person’s full motives are not identified. The person may assume that the surgical intervention will address all their limitations and allow them to return to their pre hallux valgus state, however, this is not possible since surgery can only reduce the deformity.

Defining surgical goals improves the quality of care [39]. Setting goals according to the SMART (specific, measurable, acceptable, realistic, time-bound) criteria [40] facilitates the achievement of realistic expectations, however is not easy during a short surgical consultation. More than 139 self-report questionnaires relating to the ankle and foot have been published [41, 42] but none allow a comprehensive, precise and pertinent evaluation of the foot and ankle that could facilitate goal setting. Furthermore, they do not cover the 4 main expectations of individuals for hallux valgus surgery that have been reported in the literature: pain, footwear, physical activities and aesthetics [7, 43–45]. A specific tool that the surgeon can use to determine the individual’s motives for and expectations from hallux valgus repair is therefore required.

The aim of this study was to develop a questionnaire that would encompass the main expectations of hallux valgus repair that could influence satisfaction with postoperative outcomes. To this purpose, we used the Delphi method [46]. This method uses rounds of questionnaires to collate expert opinion on specific subjects until consensus is reached [47, 48].

## Method

### Design

We used an inverse approach to develop a questionnaire relating to expectations from the reasons for dissatisfaction with hallux valgus repair: the reasons for dissatisfaction were collected from medical records for the development of the initial questionnaire. We then applied the Delphi method in 4 rounds to reach a consensus-based questionnaire.

The study is reported according to the Standards for Reporting Qualitative Research guidelines [49]. The study was approved by the Committee for The Protection of Persons Sud-Est III (reference RCB 2021-A00603-38, CPP 2021-034B). Written consent was obtained from all participants.

### Review of reasons for dissatisfaction with hallux valgus repair

In 2015, we created a system of postoperative consultations specifically for individuals who were insufficiently satisfied with the outcomes of forefoot surgery. All the consultations were conducted by 2 podiatrists who were not involved in pre-surgical or surgical care. The podiatrists recorded both the subjective and objective reasons for dissatisfaction in the person’s medical file. The purpose of the consultation was to determine possible solutions to improve satisfaction.

We collected all the reasons for dissatisfaction recorded in the medical files between 2015 and 2021 and collated them to determine reasons that were recurrent. We also compared the reasons for dissatisfaction recorded by the podiatrists with the motive for repair recorded by the surgeon.

The list of recurrent reasons was submitted to the steering committee who grouped the reasons into 4 main types of expectations (pain, footwear, aesthetics and physical activity), according to reports in the literature [41–43, 45]. Using these data, they then drafted 52 questions that constituted the initial questionnaire (S1 File).

### Steering committee

The steering committee was composed of 2 podiatrists from the La Croix Saint-Simon hospital, France’s leading hospital for orthopedic foot surgery, and 2 orthopedic surgeons from the national healthcare system who were experts in foot surgery.

### Expert panel

We selected francophone experts (from France, Belgium and Canada) by screening the lists of members of relevant academic societies: we verified their curriculum vitae and asked those who had at least 5 years’ experience in the treatment of hallux valgus and who had published or communicated on the subject to participate, according to the recommendations for conducting Delphi surveys [50]. They all provided written consent for participation and signed confidentiality forms.

Of the 40 experts contacted, a total of 34 agreed to participate: 27/34 (79.4%) men and 7/34 (20.6%) women. Their professions were orthopaedic surgeon (27/34; 79.4%), rheumatologist (2/34; 5.9%), podiatrist (5/34; 14.7%). Mean age was 50.4 (SD 9.6) years, mean duration of practice was 20.8 (10.6) years and mean experience in the management of foot deformities was 20.3 (9.3) years. The areas of expertise of the panel members are shown in Table 1.

**Table 1:** Professional activity and areas of expertise of the members of the expert panel.

Professional activity	n (%)
Hospital	11 (32)
Private structure	15 (44)
Mixed	8 (24)
Total	34 (100)
Type of department	n (%)
Orthopaedic and trauma surgery	29 (85)
Rheumatology	2 (6)
Podiatry	2 (6)
Diabetes	1 (3)
Total	34 (100)
Type of specialist consultation	n (%)
Foot and ankle	19 (56)
Knee and hip	8 (23)
Sports medicine	1 (3)
Podiatry	5 (15)
Diabetic foot	1 (3)
Total	34 (100)

The experts’ academic degrees were PhD (4/34; 11.8%) and MSc (10/34; 29.4%). The remaining experts had no postgraduate academic degree (20/34; 58.8%).

In total, 4/34 (11.8%) had no postgraduate certificates and 30/34 (88.2%) had at least 1 post graduate certificate (range 1 to 11 certificates).

Most of the experts were involved in research: 11/34 (32.4%) had published between 1 and 5 articles and 15/34 (44.1%) had published more than 5; 30/34 had presented at conferences (88.2%). Self-report of their knowledge of hallux valgus surgery was 6.1 (SD 0.8) on a Likert scale of 1 to 7.

### Delphi method

Developed in 1950 by Olaf Helmer at the Rand Corporation [46], the purpose of the Delphi method is to converge the opinions of a group of experts to create consensus on specific subjects through rounds of questionnaires [47]. This method involves a structured communication process with a defined group of experts and is based on the principle of individual and anonymous exchanges. The purpose for the present study was to identify and refine the most pertinent items for our measurement tool [48].

**Fig 1:**
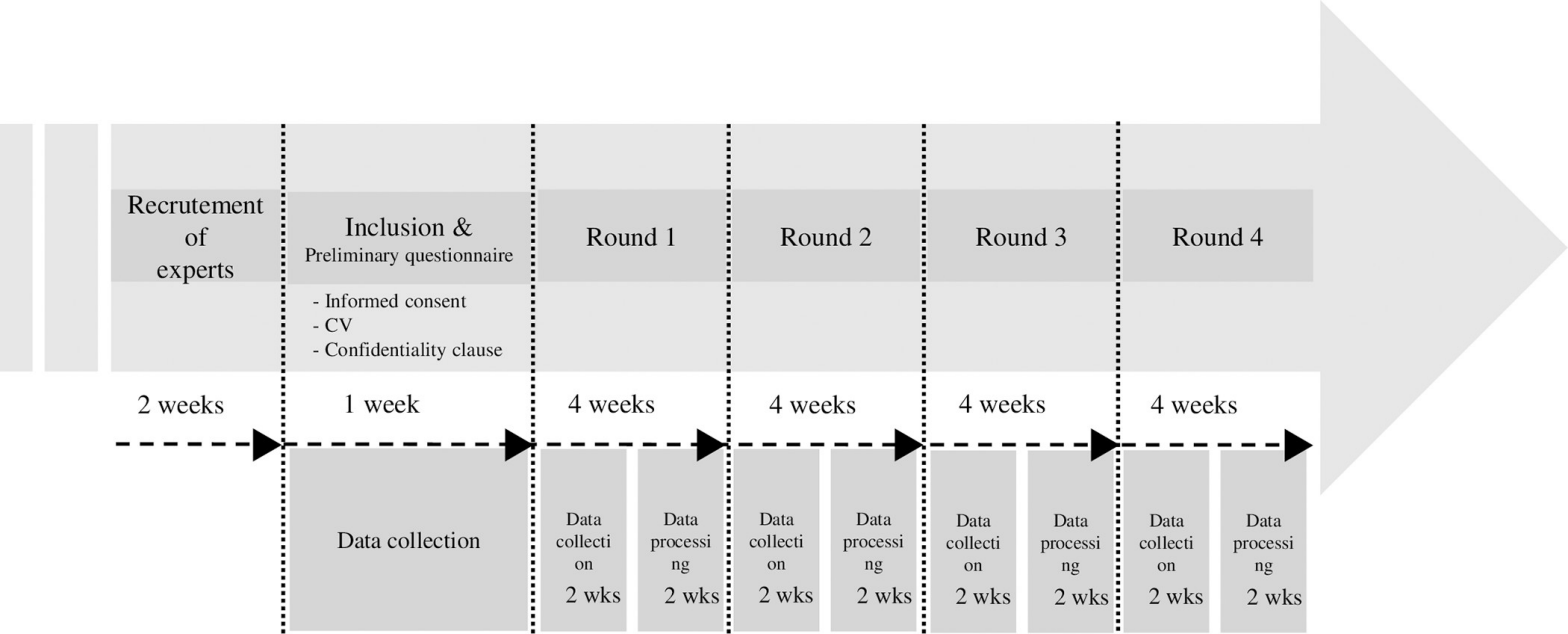
Study schedule for the DELPHI method.

**Fig 2:**
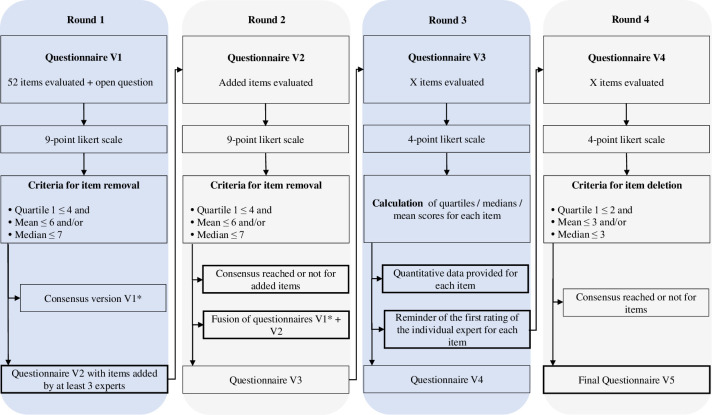
Item selection criteria for the DELPHI method.

Four rounds of questionnaires were conducted between November 2021 and January 2022 (Fig 1). At each stage, the questionnaire was submitted to the expert panel via the Drag’n Survey tool (RGPD compatible). It was modified between each stage by the steering committee, according to the comments from the expert panel. No personal data were collected online. The criteria for selection of the consensus-validated items are presented in Fig 2.

### Delphi round 1

In the first round, the initial questionnaire (V1) was submitted to the expert panel. They were asked to rate each item according to the degree of importance of the item in their opinion using a 9-point Likert-type scale [51] ranging from ’strongly disagree’ (score = 1) to ’strongly agree’ (score = 9). An open-ended question provided at the end asked the experts to provide any comments, add items they felt were missing or rephrase items if they felt it was necessary in order to ensure the comprehensibility of each question and the possible answers of the individuals completing it.

We then selected the questionnaire items according to the ratings [52]. An item was removed if 25% of the experts rated it ≤ 4 (1st quartile score) and if the mean score was ≤ 6 and/or the median score was ≤ 7. The items retained constituted the V1* questionnaire.

Additional items suggested by the experts in response to the open-ended question were retained if they were suggested by at least 3 (questionnaire V2).

### Delphi round 2

The second round followed the same process as the first but involved only the 3 items that were added or modified by the panel during round 1 (questionnaire V2).

After validation by the experts, the items of the V2 questionnaire were added to the V1* questionnaire to create questionnaire V3.

### Delphi round 3

In the third round, the experts evaluated questionnaire V3. A 4-point Likert-type scale was used: 1 for "strongly disagree", 2 for "disagree", 3 for "agree" and 4 for "strongly agree". The reason for the change in scale was to force the experts to decide between agreement or disagreement for each item. The results of this round were used to create questionnaire V4.

### Delphi round 4

In the fourth round, the experts were provided with the statistical parameters of position (median, mean) and dispersion (interquartile range) of the ratings of the whole expert panel for each item in V3 as well as their own first rating of each item so that they could compare their responses with those of the rest of the panel. The 4-point Likert-type scale was again used for the rating of each item.

Items that did not reach consensus were discarded, i.e. those with a score with a 1^st^ quartile ≤ 2 (indicating that 25% of experts disagreed), and a mean score ≤ 3 and/or a median score ≤ 3.

The retained items constituted the final version (V5).

### Statistical analysis

We described continuous and ordinal variables by their position (mean, median) and dispersion (standard deviation, interquartile range) and categorical variables by their distribution.

The analyses were carried out using R software version 4.1.0.

## Results

### Reasons for dissatisfaction with surgical outcomes

Of the 6080 individuals who underwent hallux valgus repair between 2015 and 2021, 853 (14.0%) consulted our unit because of dissatisfaction. Comparison of the reasons for dissatisfaction with the motives for repair revealed that 100% of the individuals had not fully expressed their motives at the initial consultation.

The subjective expectations that were not fully expressed during the pre-surgical consultation and that were not resolved by the repair related to footwear issues (309/853; 36.2%), aesthetic appearance (160/853; 18.8%), functional limitations (127/853; 14.9%) and pain in the foot outside the first ray (257/853; 30.1%).

### Results of the questionnaire rounds

**Fig 3:**
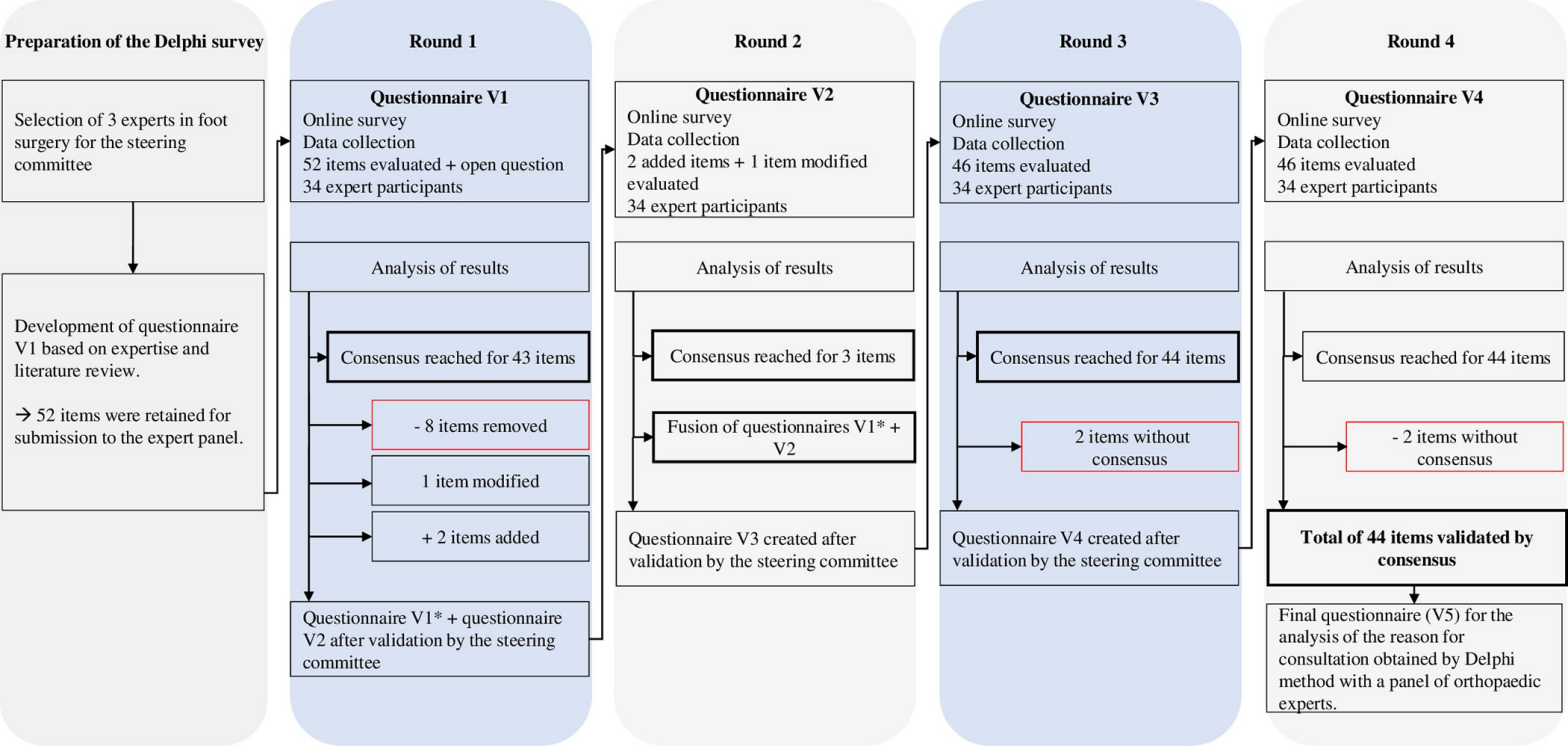
Process for obtaining consensus with the DELPHI method.

The process used to reach the final version of the questionnaire is shown in Fig 3.

In round 1, 8 items were removed (height, weight, shoe size, family history of deformity, history of sprains, sprain rehabilitation, fracture rehabilitation, daily use of stairs) (Table 2). The item regarding wearing safety shoes was reworded. The experts proposed 18 additional items for inclusion in the questionnaire, of which 2 were added.

**Table 2:** Selection of items in the Delphi method.

Items discarded after round 1			
Item	Ratings by the expert panel		
	Median	Quartile 1	Mean
Height	2.5	1.25	3.9
Weight	6.5	4.0	5.8
Shoe size	3.0	2.0	4.0
Family history of deformity	5.0	3.0	5.7
History of sprains	5.0	2.0	4.7
Rehabilitation for sprains	5.0	2.25	5.0
Rehabilitation for fractures	5.0	3.0	4.9
Daily stair use	6.0	3.25	5.8
**Items added after round 2**			
	Median	Quartile 1	Mean
Pain location	9.0	9.0	8.8
Anxiety	8.0	5.3	6.9
Special shoes at work	9.0	8.0	8.2
**Items at risk of being discarded after round 3**			
	Median	Quartile 1	Mean
History of fractures	3.0	2.0	2.9
Stairs during physical activity	3.0	2.0	2.9
**Items discarded after round 4**			
	Median	Quartile 1	Mean
History of fractures	3.0	2.0	2.8
Stairs during physical activity	3.0	2.0	2.9

Ratings on a 9-point Likert-type scale for rounds 1 and 2 and a 4-point Likert-type scale for rounds 3 and 4.

In the round 2, the 2 previously added items (location of pain, patient anxiety) and the modified item (wearing shoes at work) were validated (Table 2).

In round 3, 2 items met the conditions for elimination in the final stage if the rating was maintained in the following round (history of fractures and use of stairs during physical activity) (Table 2).

During round 4, 14.3% (224/1564) of the responses were modified because the experts changed their opinion.

The items with the highest frequency of change of opinion related to:

- hallux pain due to skin lesions (9/34; 26.5% change of opinion)
- daily stair use (9/34; 26.5% change of opinion)
- self-reported hallux valgus deformity in the right foot (8/34; 23.5% change of opinion) and left foot (7/34; 20.6% change of opinion)
- asymmetry between the feet considered unsightly by the individual (8/34; 23.5%).
- The two items eligible for elimination in the previous stage were definitively eliminated (Table 2).

In total, 44 items were retained, of which 13 related to the individual’s clinical and psychological profile, 5 to pain, 9 to physical and functional discomfort, 4 to aesthetic appearance and 13 to footwear and use of orthopaedic devices.

## Discussion

To our knowledge, this is the only study to have attempted to develop a tool to identify patient expectations during a pre hallux valgus repair consultation to improve satisfaction with postoperative outcomes. The study is original in that it involved an inverse approach that began with the reasons for dissatisfaction with hallux valgus repair of 853 individuals. The reasons for dissatisfaction were grouped into 4 themes according to the literature (pain, footwear, aesthetics and physical activity), and a 52-item questionnaire was drafted. The pertinence of the items was then improved by a 4-round Delphi approach which resulted in the final 44 item questionnaire.

### Expert panel

We chose to include only French-speaking clinicians in the expert panel since our aim was to design a questionnaire for use in France. We included a high proportion of orthopaedic surgeons (79.4%) because these professionals are the most often consulted preoperatively. However, we also included other types of clinicians because individuals who are dissatisfied with surgical outcomes do not always return to their surgeon but may prefer to seek advice from other types of foot expert.

The mean age (50.4, SD 9.6 years), number of years of practice (20.8, 10.6 years) and experience with foot deformities (20.3, 9.3 years) of the expert panel demonstrated that they were indeed experts. They kept their knowledge up to date through post graduate courses, attending symposia about hallux valgus management, and as members of learned societies. In addition, the majority (77%) of experts had published in this domain.

### Reasons for dissatisfaction

According to the literature, individuals seeking hallux valgus repair expect an improvement in pain, aesthetics, footwear or function [7, 53]: this corresponded to the reasons for dissatisfaction found in our review of medical records (S2 File). Studies have related dissatisfaction with hallux valgus repair to post-surgical complications and have reported rates of dissatisfaction between 10.6 and 33% depending on the surgical technique used [31, 37, 54, 55]. Therefore, the 14.0% rate of dissatisfaction in the present study is within the range of other studies, however our results show that the dissatisfaction was not caused by complications, but by a lack of achievement of expectations.

The results of the review of dissatisfaction showed a 30.1% rate of post repair pain, which is similar to the 31% rate in the literature; furthermore, pain mainly affected the forefoot but not the 1^st^ ray, which corresponds to the location reported in the literature [37, 56].

We found that 36.2% of individuals were dissatisfied post repair because of footwear issues. One study found that foot width did not change after surgery in 37% of cases and that it actually widened in 18% of cases [57]. Another study found that 14% of individuals still experienced discomfort even with comfortable footwear post repair [58]. Together with our results, our findings support the suggestion by Robinson et al. [58] that preoperative counselling is essential to ensure realistic expectations from repair.

Hallux valgus has been found to limit function in 15% of affected individuals [59], however, repair improves function in most cases [32]. Our results agree with this in that 85% of the dissatisfied individuals were not dissatisfied with the functional outcomes of the repair. However, 15% expected to achieve a higher level of functional improvement post repair than that which occurred, and this was the cause of their dissatisfaction: again, this demonstrates the importance of a preoperative discussion about the goals of surgery.

The American Orthopedic Foot Ankle Society Board of Directors published a statement that hallux valgus surgery should not be performed for aesthetic purposes [60]. As a result, there is a lack of data relating to the impact of repair on aesthetic outcomes with which to compare the rate of dissatisfaction of the individuals in the present study. According to the literature, individuals consider the aesthetic results of repair as good/excellent in 88% to 96% of cases: this is based on subjective perception of 1st ray alignment and the visual appearance of the scar [61–63]. The higher rate of dissatisfaction in the present study (19%) could be explained by other aesthetic considerations, such as asymmetry in the morphology of the two feet.

These results show that dissatisfaction may not only be related to complications as reported in the literature [37] but to an expression of expectations that is only partial. A typical example is problems with footwear: the individual explains to the surgeon that they want surgery to resolve their footwear problems. The surgeon assumes that the problems are caused by rubbing of the exostoses, which they remove during surgery. However, the person is not fully satisfied because what they really wanted was to be able to wear their tango shoes from the 1980’s again. Such personal goals may be difficult to ascertain during a consultation [53]: this supports the development of a tool to facilitate the identification of individuals’ expectations from hallux valgus repair.

### Development of the questionnaire and validation of items by the expert panel

Of the 52 items in the initial questionnaire developed by the steering committee from the reasons for dissatisfaction of the 853 individuals, 44 were validated by the expert panel.

Among the general questions, age and sex were retained. Hallux valgus is more frequent in women than men and in older than younger people [4, 58], furthermore, women tend to have greater difficulties with shoe fitting because of hallux valgus than men. Although age does not appear to have an impact on surgical outcomes [64, 65], older people tend to have a more sedentary lifestyle than younger people [66] and may therefore have different functional expectations that should be considered in the surgical decision.

The first item that related to expectations involved ranking the motives for consultation in their order of importance to the individual (pain, footwear, physical activity and aesthetics). This item achieved the full consensus of the panel. None of the terms were modified, which demonstrates that they encompass the main motives for repair according to the panel’s experience. The following item asked about the time expected by the individual for improvement to occur. This also achieved full consensus. The individual must be aware of the healing time to avoid increases in function or the wearing of certain types of footwear too rapidly, which could risk damaging the repair [67].

The items relating to pain were all validated by the expert panel; the experts also added an item relating to pain location. This makes sense since hallux valgus may cause pain in areas other than the first metatarsophalangeal joint, such as the lateral metatarsals [27]. A precise analysis of the extent of preoperative pain is essential since the degree of preoperative pain may influence postoperative persistence of pain [56]. Furthermore, metatarsal pain can arise secondary to hallux valgus surgery [37, 68]: a thorough identification of pain areas may therefore limit dissatisfaction arising from poor identification of preoperative pain.

The panel validated all the items relating to aesthetics, confirming reports in the literature that many individuals seek repair to improve the aesthetic appearance of their foot [43, 45, 69]. Furthermore, aesthetic perceptions may influence pain levels and the functional benefits of surgery [69]. Since hallux valgus repair should not be performed for cosmetic reasons [59], such motives must be ascertained preoperatively.

All the items that concerned physical activity, daily life and occupation were validated, except for stair-use. Resuming previous activities, including sports may be an important motive for hallux valgus repair for some individuals [70]. Therefore, the identification of specific functional objectives is very important to ensure that they are realistic. Hallux valgus repair may also require a period of sick leave [71], therefore the person must be aware of the potential duration of their recovery to plan accordingly.

Some individuals want hallux valgus repair so they can wear standard or specific types of footwear [58]. This obviously corresponded with the panel’s experience since they validated all items relating to footwear and expanded the item relating to footwear at work to encompass all types of footwear. Studies have shown that hallux valgus repair improves foot morphology [57, 72] and shoe fitting [58], however, it is not always possible to return to wearing all kinds of shoes (e.g. high heels) postoperatively. Expectations and likely outcomes should therefore be thoroughly discussed prior to surgery.

Many individuals with hallux valgus use orthotic devices. The results of our review of the reasons for dissatisfaction showed that some individuals were disappointed if they still required an orthotic device post-surgery, particularly if they had not been informed of this preoperatively (S2 File). All the items relating to orthotic devices were validated by the expert panel. This section of the questionnaire should be useful to determine if orthotics have not been attempted: they could be tried as they may avoid the need for surgery, particularly if the person’s motive is to reduce pain [73, 74].

### Discarded items

Eight items that were included in the initial questionnaire were discarded by the expert panel. Among the general items, weight and height were discarded: these variables are unlikely to influence the motive for hallux valgus repair [75–77] and were thus unnecessary. In addition, these data are systematically collected before the consultation as they are part of the medical file.

We were surprised that the items relating to shoe size and family history of deformity were discarded since these issues were frequent reasons for dissatisfaction (S2 File). They may be considered to be related to the aesthetic aspects of hallux valgus, and therefore not indicative of a surgical procedure, which may explain the choice of the panel who found these items not useful [58].

A history of ankle sprain or fracture was a source of post-repair dissatisfaction for some of the individuals. If some instability remained, it could be exacerbated by the anatomical changes caused by the repair [78–80]. However, to our knowledge, no such reports of increased instability post repair exist in the literature, which may explain why the experts removed this item.

We included items about stair-use because pain on stairs was a complaint of a proportion of the sample. However, the experts may have considered that, in contrast with hallux rigidus, hallux valgus deformity does not systematically limit extension of the first metatarsophalangeal joint and thus should not limit stair use [81, 82]. Furthermore, metatarsophalangeal joint arthrodesis, which might affect stair climbing, is usually only performed in the case of severe deformities or during surgical revision [83, 84].

### Additional items

Anxiety was not an issue that emerged from the review of the reasons for dissatisfaction, therefore we had not included this concept in the initial questionnaire. Although, the association between psychological symptoms or personality traits and postoperative outcomes is somewhat debated [85, 86], anxiety and depression may increase pre-surgical expectations, levels of pain perception, and post-surgical dissatisfaction [87, 88]. The panel therefore felt that questions about anxiety relating to the individual’s personal or professional situation, the foot deformity, the surgical procedure, or what would happen if surgery was not undertaken were necessary. These questions should facilitate consideration of the psychological aspects relating to hallux valgus and its repair in treatment planning, and particularly the decision to perform a surgical intervention.

### Evaluation of hallux valgus

Although the Manchester scale has been validated against radiographic measurements and can indicate pressure thresholds related to deformity, the decision to undertake surgery cannot be based on visual observation alone [89, 90]. However, the role of radiographic findings in surgical decision making is debated. There appears to be no correlation between the severity of preoperative hallux valgus, radiographic correction of the deformity and the post-op SF 36 quality of life score [32]. Since in orthopaedic surgery, subjective, functional and objective outcomes are not always related, the results of hallux valgus surgery cannot be reduced to the radiographic outcome alone [7]. Radiological measurements can bias the interpretation of the deformity and should always be compared with the clinical examination data [24]. There are difficulties associated with defining each individual’s deformity despite imaging, and thus the optimal choice of management. The three-dimensional classification of hallux abducto valgus may be unreliable, supporting the fact that two-dimensional radiographs are limited for the assessment of three-dimensional deformity [91]. With the development of Cone beam weightbearing computed tomography, a new classification system may be necessary. The three-dimensional pattern of the deformity must be understood in order to plan surgery.

Stato-dynamic baropodometry is necessary for a functional evaluation of the foot. However, it is currently mainly used to assess post-surgical outcomes in terms of changes in plantar pressures, because criteria have not been defined for diagnosis and surgical decision making [92–96]. Studies are still need to assist diagnosis and facilitate surgical decisions.

The difficulties associated with assessment and classification of hallux valgus support our pre-surgical questionnaire to limit the risk of inappropriate decision making. The evaluation tools for hallux valgus allow a decision to be made according to the anatomical relevance of the surgical procedure to be carried out, but do not evaluate the relevance in relation to the individual’s expectations for their life after surgery. The biomechanics of this three-dimensional deformity are extremely complex and understanding the individual’s expectations is a challenge.

The questionnaire validated by the Delphi method in the present study could be completed prior to a consultation for surgical advice, without encroaching on the practitioner’s time. Further studies are now needed to validate its clinical utility for individuals with hallux valgus.

## Limitations

We did not investigate possible external influences (social media and marketing) on the individual’s decision to undergo hallux valgus surgery.

We also did not consider the individual’s history of medical/paramedical consultations for the hallux valgus deformity or the possible etiologies of the deformity (family history, congenital aspect, etc.). as this information should be documented in the medical file.

Although this questionnaire is intended to limit the risk of surgical decision-making being influenced by personal factors or medical jargon used by practitioners, it will not prevent an unbalanced caregiver-patient relationship.

## Conclusion

Using data relating to dissatisfaction with hallux valgus repair and the Delphi method, we developed a 44-item questionnaire to determine individuals’ expectations from surgical repair. We believe the use of this questionnaire will reduce dissatisfaction with postoperative outcomes by ensuring that surgical objectives and methods match expectations. Further studies are now required to evaluate the psychometric properties of this tool, and to create a scoring method that would allow surgeons to rapidly determine if the individual’s expectations are appropriate. The next step is to validate this questionnaire in a large sample of individuals with hallux valgus to evaluate the benefits of supporting individuals to discern their reason for consultation. Following this, clinical trials should assess the effect of use of the questionnaire on post operative satisfaction.

## Supporting information

S1 File Questionnaire to determine the reasons for consultation. (PDF)

S2 File Main subjective expectations that were not fully expressed during the pre-surgical consultation and not resolved by hallux valgus repair. (PDF)
