# Supplementary material for: Development of a preoperative questionnaire to improve satisfaction with hallux valgus repair: A Delphi study
Source: PLoS One. 2022 Oct 24;17(10):e0276303. doi: 10.1371/journal.pone.0276303 (PMC9591061; doi:10.1371/journal.pone.0276303)
Supplement: S1 File — (PDF) [file pone.0276303.s001.pdf]

It is important to note that this questionnaire was conducted in French. It has been translated here, however has not undergone cross-cultural validation.

Questionnaire to determine the reasons for consultation

Items in red were discarded, those in blue were added and those in green were modified during the Delphi process.

▪ GENERAL QUESTIONS

Q1 Are you:

• Male • Female

Q2 How old are you:

Response: ………. years

Q3 How tall are you:

Response: ………. cm

Q4 How much do you weigh:

Response: ………. kg

Q5 What is your shoe size:

Response: ……….

Q6 You have a deformity of your right big toe (hallux valgus).

• Yes • No • I don't know

Q7 You have a deformity of your left big toe (hallux valgus).

• Yes • No • I don't know

Q8 How old were you when you noticed the deformity?

Right foot: ………. years Left foot: ………. years

Q9 Does one of your parents or grandparents have a similar deformity of their big toe?

• Yes • No • I don't know

Q10 Classify in order of importance (from the most to the least important), why you are consulting for foot surgery:

A) Pain around my big toe B) The aesthetic appearance of my foot

C) Problems with footwear pressing/rubbing D) I am limited in my daily activities

Example: D > C > B > A
In this example, the most important reason for consultation is limitation of daily activities; the least important reason for consultation is pain.

Your response: ……… > ……… > ……… > ………

Q11 After hallux valgus surgery, how quickly would you like to see results that meet your expectations?

• 3 - 6 months • 6 – 12 months • More than 12 months • Other: ………….

▪ QUESTIONS ABOUT PAIN

*Q11** Your pain is located:

• On the big toe deformity • On the little toes • Under the foot • I have no pain

Q12 You have pain in your big toe at night

• Yes, on the right foot • Yes, on the left foot • No

Q13 You have pain in your big toe when you put weight on it

• Right foot		• Left foot	
Barefoot	yes/no	Barefoot	yes/no
With shoes	yes/no	With shoes	yes/no

Q14 Your big toe hurts because it presses/rubs in your shoe?

• Yes, the right foot • Yes, the left foot • No

Q15 You have pain in your big toe because of skin lesions (corns, calluses).

• Yes, the right foot • Yes, the left foot • No

■ QUESTIONS ABOUT FOOTWEAR

Q16 You need to take one or more extra shoe sizes because of your big toe deformity.

• Yes • No

Q17 You have had to stop wearing high heeled shoes (> 3cm) because of your big toe deformity.

• Yes • No • I don't wear high heels

Q18 You have to wear special shoes (bought in a pharmacy) because of your big toe deformity.

• Yes • No

Q19 You have to wear open shoes because of your big toe deformity.

• Yes • No

Q20 You have to wear sports shoes (trainers) because of your big toe deformity.

• Yes • No

Q21 You have to wear safety shoes at work. (See Q43*).

• Yes • No

Q22 You want hallux valgus surgery because you hope to be able to wear shoes that you can't wear just now again.

• Yes • No

■ QUESTIONS ABOUT AESTHETIC APPEARANCE

Q23 You find your big toe deformity ugly.

• Yes, on the right foot • Yes, on the left foot • No • I don't have an opinion

Q24 You find your other deformed big toes ugly.

- Yes, on the right foot
- Yes, on the left foot
- No
- My other toes are not deformed
- I don't have an opinion

Q25 You find the difference in shape between your left and right feet (asymmetry) ugly.

- Yes • No • I don't have an opinion • My feet are identical.

Q26 You want hallux valgus surgery because you hope that your foot will look the same as the other one.

- Yes • No

▪ QUESTIONS ABOUT ORTHOTICS

Q27 You wear custom made orthopaedic insoles because of your big toe deformity.

- No, I have never worn any
- Yes, I am still wearing them
- Yes, but I stopped using them

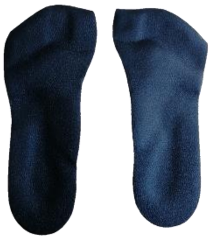

Q28 You wear standard insoles (bought in a pharmacy or supermarket) because of your big toe deformity.

- Yes • No

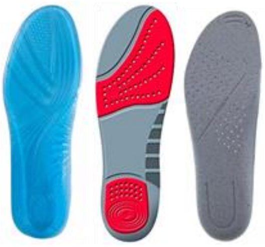

Q29 You wear toe separators (bought in a pharmacy or shop) because of your big toe deformity.

- Yes • No

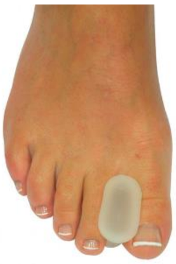

Q30 You wear custom made toe separators because of your big toe deformity.

• Yes • No

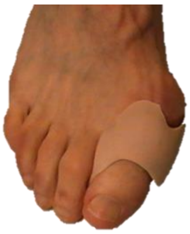

Q31 You wear gel pads under your forefoot because of your big toe deformity .

• Yes • No

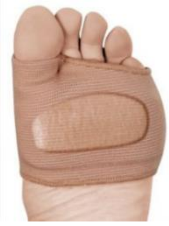

Q32 You wear skin protectors because of your big toe deformity.

• Yes • No

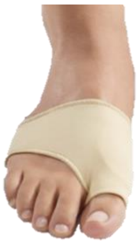

▪ YOUR HISTORY:

Q33 Have you ever sprained your ankle or foot?

• Yes, the right one • Yes, the left one • No

Q34 If yes, did you have any rehabilitation for the sprain(s)?

• Yes, for the right one • Yes, for the left one • No

Q35 Have you ever fractured your ankle or foot?

• Yes, the right one • Yes, the left one • No

Q36 If yes, did you have any rehabilitation for the fracture?

• Yes, for the right one • Yes, for the left one • No

*Q36** Are you anxious?

• Yes, about my foot problem
• Yes, about the fact I might have surgery
• Yes, for personal/professional reasons
• Yes, about what will happen to my foot if I don't have an operation
• No

▪ YOUR PHYSICAL ACTIVITY:

Q37 During the last 3 months, how many hours of physical activity did you do on average per week? Specify which types.

Response:
………………………………………………………………………………………………

Q38 You can practice moderate physical activity (1 h/week).

• Yes
• No, because of my hallux valgus
• No, for other medical reasons
• I don't do any physical activity

If yes, have you practiced any physical activity during the past 3 months?
• Yes • No

Q39 You can practice intense physical activity (3 h/week).

• Yes
• No, because of my hallux valgus
• No, for other medical reasons
• I don't do any physical activity

If yes, have you practiced any physical activity during the past 2 weeks?
• Yes • No

Q40 You can practice impact sports: running, ball games, dancing etc.

• Yes
• No, because of my hallux valgus
• No, for other medical reasons

• I don't do any physical activity

Q41 You can go up and down stairs several times a day.

• Yes
• No, because of my hallux valgus
• No, for other medical reasons
• I don't use stairs

▪ YOUR DAILY LIFE:

Q42 Your job involves a lot of standing.

• Yes • No • I don't work • I am on sick leave.

Q43 You drive:

• Every day
• Several times a week
• Several times a month
• Never
• Every day because it is part of my job.

Q43* At work it is mandatory that you to wear special shoes:

• Yes • No (Modification of Q21).

Q44 You walk up and down stairs (at least one floor).

• Every day • Several times a week • Several times a month • Never

▪ YOUR FUNCTIONAL GOALS:

Q45 Following surgery, what activities do you absolutely want to be able to continue doing? (Example: dancing, yoga, hiking, climbing stairs, kneeling etc.)

Response:
……………………………………………………………………………………………

Q46 During the past 3 months how many times did you perform these activities?

Response:
……………………………………………………………………………………………

Q47 After surgery, how soon do you think you will be able to resume these activities??

• 3 - 6 months • 6 – 12 months • > 12 months • Other: ………………….

Q48 Which of the activities that you are currently still able to do but that cause pain would you be willing to give up?

Response:
…………………………………………………………………………………………………
……………………………………………………………

Q49 Following surgery, is it your aim to return to an activity that you can no longer do because of your hallux valgus?

• Yes • No

If yes, which one?
Response:
…………………………………………………………………………………………………

▪ OTHERS:

Q50
After reading this questionnaire, you realised that you had not asked yourself certain questions.

1. Completely disagree. 2. Disagree. 3. Agree. 4. Completely agree.

Q51
You feel that these questions are important in understanding your big toe problem.

1. Completely disagree. 2. Disagree. 3. Agree. 4. Completely agree.

Q52
This questionnaire will have an impact on your decision to undergo surgery.

1. Completely disagree. 2. Disagree. 3. Agree. 4. Completely agree.
