# Supplementary material for: Development of a preoperative questionnaire to improve satisfaction with hallux valgus repair: A Delphi study
Source: PLoS One. 2022 Oct 24;17(10):e0276303. doi: 10.1371/journal.pone.0276303 (PMC9591061; doi:10.1371/journal.pone.0276303)
Supplement: S2 File — (PDF) [file pone.0276303.s002.pdf]

Main subjective expectations that were not fully expressed during the pre-surgical consultation and not resolved by hallux valgus repair

	n
**Footwear issues**	**309**
Inability to wear certain shoes that were used in the past (even many years ago)	77
Inability to wear certain high-heeled shoes	66
Inability to wear certain narrow shoes	72
Inability to wear mandatory work shoes	11
Inability to wear shoes of a certain size	33
Skin lesions with certain shoes	32
Adaptation of footwear required (comfortable shoes)	18
**Aesthetic appearance**	**160**
Foot size	21
Hyperkeratosis	17
Morphological asymmetry between the feet (toe length, foot width, scar)	45
Alignment of the hallux	36
Alignment of lateral toes	31
Perception of unsightly shape of the foot similar to another family member	10
**Functional limitations**	**127**
Unable to resume sports during the first weeks post-surgery	25
Unable to resume activities that requiring a large amplitude of extension of the first metatarsophalangeal joint (yoga, climbing, mountain hiking, boxing, jumping rope, dancing, etc.) occasionally, moderately or intensely	26
Unable to resume certain impact sports (running, ball sports, etc.) occasionally, moderately or intensely	29
Unable to resume a previously abandoned sport	13
Unable to go up and down stairs repeatedly on a daily basis	10
Unable to stand for a length of time	4
Unable to drive a vehicle	6
Post-surgical forefoot instability due to ankle joint instability	8
Post-surgical forefoot instability due toy history of foot fractures	6
**Pain in the foot outside the first ray**	**257**
Metatarsalgia of the lateral rays during walking	176
Metatarsalgia of the lateral rays during certain movements	25
Still requires orthopaedic insoles for metatarsalgia (wore insoles pre-surgically)	56

n= number of individuals who reported the expectation
